# Evidence of health benefits of canola oil

**DOI:** 10.1111/nure.12033

**Published:** 2013-05-02

**Authors:** Lin Lin, Hanja Allemekinders, Angela Dansby, Lisa Campbell, Shaunda Durance-Tod, Alvin Berger, Peter JH Jones

**Affiliations:** Richardson Centre for Functional Foods and Nutraceuticals, Departments of Food Science and Human Nutritional Sciences, University of ManitobaWinnipeg, Manitoba, Canada; U.S. Canola AssociationWashington, DC, USA; Canola Council of CanadaWinnipeg, Manitoba, Canada; Department of Food Science & Nutrition, University of MinnesotaSt. Paul, Minnesota, USA

**Keywords:** canola oil, cardiovascular disease, human intervention studies, rapeseed

## Abstract

Canola oil-based diets have been shown to reduce plasma cholesterol levels in comparison with diets containing higher levels of saturated fatty acids. Consumption of canola oil also influences biological functions that affect various other biomarkers of disease risk. Previous reviews have focused on the health effects of individual components of canola oil. Here, the objective is to address the health effects of intact canola oil, as this has immediate practical implications for consumers, nutritionists, and others deciding which oil to consume or recommend. A literature search was conducted to examine the effects of canola oil consumption on coronary heart disease, insulin sensitivity, lipid peroxidation, inflammation, energy metabolism, and cancer cell growth. Data reveal substantial reductions in total cholesterol and low-density lipoprotein cholesterol, as well as other positive actions, including increased tocopherol levels and improved insulin sensitivity, compared with consumption of other dietary fat sources. In summary, growing scientific evidence supports the use of canola oil, beyond its beneficial actions on circulating lipid levels, as a health-promoting component of the diet.

## Introduction

Canola is a bright, yellow-flowering plant belonging to the Brassicaceae family.^1^ This family includes three different species: *Brassica napus, B. rapa*, and *B. juncea*.^1^ Originally from the Mediterranean area and Northern Europe, *B. napus* is commonly known as rapeseed. Rapeseed was identified in 2000 BC as a high-erucic acid crop, containing >40% erucic acid in the oil. Due to concerns over erucic acid content that stemmed from animal studies, high-erucic acid rapeseed oil used to be produced in North America solely in small quantities for industrial, nonfood use.^2^,^3^ In 1976, however, Canadian scientists were able to improve the quality of previous cultivars of rapeseed through traditional plant breeding, which led to a conversion to commercially consumable canola cultivars.^4^,^5^ In 1979, Canada registered the word “canola” to describe a new seed found to be oil, which was low in erucic acid and low in glucosinolates.^4^ By definition, canola has specific cut-off levels of erucic acid (<2%) and glucosinolates (<30 umol/g)^4^ for both human and animal consumption. In 1977, the low-erucic acid rapeseed (LEAR) oil containing <5% erucic acid and low glucosinolates was introduced as an edible oil in Europe.^6^ In 1985, the U.S. Food and Drug Administration granted canola oil “generally recognized as safe” (GRAS) status as a dietary component.^7^ Throughout this review, the term “canola oil” is used to generically refer to the presently available conventional canola oil in North America and conventional LEAR oil in Europe. Other types of canola oil or LEAR are specifically noted.

Over the past 40 years, canola has become one of the most important oilseed crops worldwide. Today, canola oil is the third largest vegetable oil by volume after palm and soybean oil.^3^ The worldwide production of canola oil in 2010/2011 was 38 million metric tonnes, with Europe accounting for 63% and Canada accounting for 31% globally.^8^ In the United States, canola oil is one of the most widely consumed oils, second only to soybean oil.^9^

Canola oil is characterized by the following: low level (7%) of saturated fatty acids (SFAs); substantial amounts of monounsaturated fatty acids (MUFAs) and polyunsaturated fatty acids (PUFAs), including 61% oleic acid, 21% linoleic acid, and 11% alpha-linolenic acid (ALA)^2^,^10^; plant sterols (0.53–0.97%); and tocopherols (700–1,200 ppm)^11^,^12^ – all of which have data indicating they are cardioprotective substances. With regard to the high MUFA content of canola oil, Kris-Etherton et al.^13^,^14^ and Gillingham et al.^15^ have provided evidence supporting positive effects of MUFAs compared with SFAs on cardiovascular health through the regulation of plasma lipids and lipoproteins, susceptibility of low-density lipoprotein (LDL) oxidation, and insulin sensitivity. Also, for the treatment of existing cardiovascular disease, canola oil has been recommended for achieving daily n-3 FA requirements of 1 g/day.^16^ In 2006, the U.S. Food and Drug Administration authorized the following qualified health claim for canola oil: *“Limited and not conclusive scientific evidence suggests that eating about 1½ tablespoons (19 grams) of canola oil daily may reduce the risk of coronary heart disease due to the unsaturated fat content in canola oil. To achieve this possible benefit, canola oil is to replace a similar amount of saturated fat and not increase the total number of calories you eat in a day.”*^17^ This claim was based on the validity of total cholesterol (TC) and LDL cholesterol (LDL-C) as biomarkers for coronary heart disease (CHD).

More recently, growing numbers of studies indicate that biomarkers beyond blood lipids are beneficially influenced by canola oil consumption. The objective of this review was to conduct a literature assessment to examine the health benefits of intact canola oil, rather than focus on the effects of individual components in the oil. This approach can be considered more practical since consumers make choices among intact cooking oils for consumption. Further, this review investigated whether newer data are compatible with earlier conclusions regarding the health benefits of canola oil. The specific aim of this review, based on the most recent literature available, was to describe the effects of canola oil consumption on blood lipids, inflammation, insulin sensitivity, LDL-C oxidation, energy metabolism, and cancer compared with other dietary fat sources.

## Literature Search Methods

**Figure 1:**
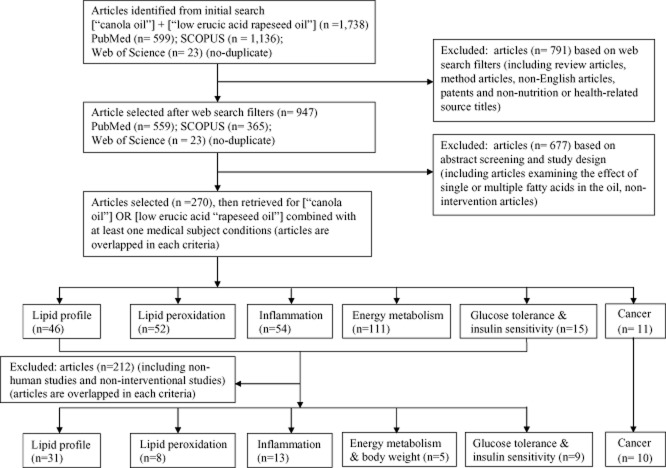
Flow diagram of article screening process for the present review.

Database searches using PubMed, SCOPUS, and Web of Science were conducted to identify nutrition-based studies published in the English-language literature with no restrictions on date of publication (Figure 1). Searches using Google Scholar were carried out to find legal documents or freely accessible articles. No studies were excluded on the basis of quality during the preliminary search phase. Initial search terms included “canola oil” OR “low erucic acid rapeseed oil.” In total, 1,738 articles were initially selected. Articles were then culled based on web filters. Review articles, method articles, non-English articles, patents, and non-health-related source titles were excluded. A total of 947 articles remained after web search filters were applied, but only 270 articles were eligible for inclusion following abstract screening and study design evaluation.

As a follow-up, articles were retrieved based on “canola oil” or “rapeseed oil” combined with 1) “lipid profile” AND “coronary heart disease”; 2) “susceptibility of low-density lipoprotein (LDL-C)” OR “peroxidation”; 3) “inflammation” OR “anti-inflammatory” OR “platelet coagulation” OR “endothelial function”; 4) “body weight” OR “energy metabolism”; 5) “insulin sensitivity” OR “glucose tolerance”; and 6) “cancer.” Pertinent articles including the search terms “glycemic control,” “blood pressure,” or “fat deposition” were then examined. In addition, the reference lists from retrieved articles were searched to ensure that no study was unintentionally excluded.

Although animal models can provide evidence of a treatment effect and are beneficial for studying human health conditions,^18^ the biological differences between the type of animal model and the bias of design in mimicking a clinical condition can cause discordant results between animal and human studies.^19^,^20^ The present review eliminated all nonhuman studies, with the exception of those related to cancer conditions; for these conditions, cell culture and animal studies were included because only limited human cancer study data exist to date.

In all, the follow-up search retrieved a total of 31 studies for lipid profile, eight for lipid peroxidation, 13 for inflammation, five for energy metabolism, nine for glucose tolerance, and 10 for cancer using the search criteria described. In some instances, studies were duplicated across topics covered. The details of each study are summarized in Tables 1 and 2, and pivotal studies within each category are described in the relevant sections of text below.

**Table 1:** Summary of studies investigating circulating blood lipid profiles with different types of diets

Reference	Study design	Canola oil treatment	Control diet	Duration of exposure	Participants	Baseline status (mmol/L)	Magnitude of effect (%)^a^	*P* value
Baudet et al. (1988)^37^	R, C, CRO	15.6 E% CO	15.6 E% milk fat	6 weeks	Females, 26–49 years, *n* = 20	TC = 5.38 LDL = 3.54 HDL = 1.78 TAG = 0.86	TC ↓ 18.4% LDL↓ 24.9% HDL ↑ 1.7% TAG ↓ 4.7%	*P* < 0.01 *P* < 0.01 NS NS
Corboy et al. (1993)^50^	C	30 g CO mix into 135 g muesli	Baseline normal diet	4 weeks	Male, 20–65 years, *n* = 8	TC = 4.57 ± 1.09 LDL = 2.90 ± 0.90 HDL = 1.13 ± 0.17 TAG = 0.94 ± 0.36	TC ↓10.5% LDL↓10.7% HDL↑ 1.8% TAG↓ 6.4%	*P* < 0.05 *P* < 0.05 NS NS
Gillingham et al. (2011)^25^	R, SB, CRO	24.5 E% high-oleic CO	1) 24.5 E% provided by a blend of oils typical of a Western diet 2) 24.5 E% flaxseed and high-oleic CO	4 weeks	Males and females, 18–65 years, *n* = 36	TC = 5.94 ± 1.03 LDL = 3.70 ± 0.95 HDL = 1.41 ± 0.35 TAG = 1.84 ± 1.09	1) TC↓6.7% LDL↓12.2% HDL↓ 2.9% TAG↑12.9% 2) TC↑2.8% LDL↑0.6% HDL↑3.76 TAG ↑ 10.3%	1) *P* < 0.001 *P* < 0.001 NS NS 2) *P* < 0.05 NS *P* < 0.05 NS
Gulesserian et al. (2002)^21^	Not applicable	Avg. 15 g/day CO during first 2 months, avg. 22 g/day CO during last 3 months	Baseline habitual diet	5 months	Males and females, 4–19 years, *n* = 17	TC = 6.03 LDL = 3.90 HDL = 1.53 TAG = 2.04	TC ↓ 8.6% LDL ↓ 6.0% HDL ↓ 3.4% TAG ↓ 28.8%	*P* < 0.05 *P* < 0.01 NS *P* < 0.05
Gustafsson et al. (1994)^26^	R, C, P	∼30 E% fat, mainly RO	Baseline: Swedish diet, 37 E% fat, 16 E% SFA	3 weeks	Males and females, avg. 48.2 ± 6.9 years, *n* = 46 (RO group)	TC = 6.79 ± 1.46 LDL = 4.85 ± 1.24 HDL = 1.12 ± 0.27 TAG = 2.08 ± 0.80	TC ↓ 15.5% LDL ↓ 19.6% HDL ↓ 10.7% TAG ↓ 13.5%	*P* < 0.001 *P* < 0.0001 *P* < 0.001 *P* < 0.01
Hodson et al. (2001)^30^	R, CRO	29 g/day CO and CO margarine, 28.9% of total fat intake	High-SFA diet: 34 E% fat, 17.7 E% SFA	2.5 weeks	Males and females, avg. 23.0 ± 4.2 years, *n* = 42	TC ≤ 6.5	TC ↓ 11.6% LDL ↓ 14.8% HDL ↓ 4.1% TAG ↓ 15.0%	*P* < 0.001 *P* < 0.001 *P* < 0.05 *P* < 0.001
Iggman et al. (2011)^38^	R, CRO	CO diet, 35 E% fat: CO-based margarine	Dairy fat diet, 35 E% fat: butter, cream and high-fat cheese	3 weeks	Males and females, 25–68 years, *n* = 20	TC = 6.70 ± 1.20 LDL = 4.76 ± 1.13 HDL = 0.98 ± 0.31 TAG = 2.21 ± 1.19	TC ↓ 16.1% LDL ↓ 19.6% HDL ↑ 1.0 TAG ↓ 12.8	*P* < 0.0001 *P* < 0.0001 NS *P* < 0.05
Junker et al (2001)^51^	R, C	38.4 E% RO diet; 19 E% MUFA	Wash-in diet 38 E% 19 E% SFA	4 weeks	Males average 25 years, *n* = 58	TC = 4.58 ± 0.78 LDL = 2.53 ± 0.79 HDL = 1.66 ± 0.54 TAG = 0.78	TC ↓14% LDL ↓ 18% HDL ↓ 12% TAG ↑ 5%	*P* < 0.001 *P* < 0.001 *P* < 0.01 NS
Karvonen et al. (2002)^39^	R, C, SB, CRO	11 g/day CO in 65 g cheese (11 g fat/1 g SFA)	65 g/day milk fat cheese (15 g fat/10 g SFA)	4 weeks	Males and females, 25–65 years, *n* = 31	TC = 6.13 ± 0.59 LDL = 4.17 ± 0.56 HDL = 1.34 ± 0.35 TAG = 1.36 ± 0.48	TC ↓ 5.1% LDL ↓ 6.4% HDL ↓ 3.0% TAG ↔ 0%	*P* < 0.001 *P* < 0.01 NS NS
Lichtenstein et al. (1993)^27^	R, C, DB, CRO	20 E% CO (30 E% fat, <7 E% SFA)	1) Baseline: Western diet, 36 E% fat, 15 E% SFA 2) Corn oil 3) OO	32 days (∼5 weeks)	Males and females, 44–78 years, *n* = 14	TC = 5.72 LDL = 3.93 HDL = 1.24 TAG = 1.21	1) TC ↓ 12.2% LDL ↓ 17.0% HDL ↓ 8.0% TAG ↑ 1.7% 2) TC ↔ 0% LDL ↑ 0.8% HDL ↔ 0% TAG ↑ 0.9% 3) TC ↓ 5.4% LDL ↓ 4.5% HDL ↓ 4.3% TAG ↓ 2.7%	1) *P* < 0.001 *P* ≤ 0.001 *P* ≤ 0.05 NS 2) NS NS NS NS 3) *P* < 0.05 NS NS NS
Matheson et al. (1996)^31^	C, CRO	Avg. 16.6 g/day CO margarine and 13.5 g/day CO (= 29.5% of total fat intake)	Control diet: butter, margarine, vegetable oils	13 weeks	Males and females, 25–50 years, *n* = 23	TC = 5.82 ± 0.68 LDL = 4.41 ± 1.09 HDL = 1.07 ± 0.25 TAG = 1.73 ± 0.62	TC ↓ 7.0% LDL ↓ 10.0% HDL ↑ 6.0% TAG ↑ 20.0%	*P* < 0.05 *P* < 0.05 NS NS
McDonald et al. (1989)^28^	R, C, CRO	28 E% CO	Baseline (7 days): Western diet 36% fat	18 days	Males, 19–32 years, *n* = 4	TC = 4.48 LDL = 2.98 HDL = 1.41 TAG = 1.03	TC ↓ 20.1% LDL ↓ 25.2% HDL ↓ 10.6% TAG ↓ 20.4%	*P* < 0.05 *P* < 0.05 *P* < 0.05 NS
McKenney et al. (1995)^42^ *Study 1*	R, C, DB, CRO	42.6 g/day CO in cookies	42.0 g/day coconut oil in cookies	6 weeks	Males and females, 47–79 years, *n* = 11	TC = 5.75 LDL = 3.85 HDL = 1.29 TAG = 1.32	TC ↓ 8.7% LDL ↓ 11.1% HDL ↓ 4.1% TAG ↑ 1.1%	*P* < 0.01 *P* < 0.05 Not reported Not reported
McKenney et al. (1995)^42^ *Study 2*	R, C, DB, CRO	42.6 g/day CO in cookies	42.0 g/day coconut oil in cookies	6 weeks	Males and females, 39–63 years, *n* = 17	TC = 5.53 LDL = 3.58 HDL = 1.32 TAG = 1.38 Lovastatin	TC ↓ 4.6% LDL ↓ 5.2% HDL ↓ 5.2% TAG ↓ 0.5%	NS NS Not reported Not reported
Miettinen and Vanhanen (1994)^45^	R, DB	50 g/day of CO mayonnaise	Baseline habitual diet	6 weeks	Males, avg. 50 ± 3 years, *n* = 18	TC = 7.1 ± 0.1 LDL = 4.5 ± 0.1 HDL = 1.3 ± 0.1 TAG = 1.8 ± 0.2	TC ↓ 9% LDL ↓ 10% HDL ↑ 9% TAG ↓ 19%	*P* < 0.05 *P* < 0.05 *P* < 0.05 *P* < 0.05
Mutalib et al. (1995)^43^	R, C	26 E% hydrogenated RO	Palm oil	8 weeks	Males, age range: unknown *n* = 43	Overall average: TC = 4.60 LDL = 3.01 HDL = 1.14 TAG = 1.12	1) TC ↓ 21% LDL ↓ 32% HDL ↑3% TAG ↑ 3%	*P* < 0.05 *P* < 0.05 *P* < 0.05 NS
Nielsen et al. (2002)^59^	R, DB, CRO	19 E% CO	1) 19 E% OO 2) 19 E% SO	3 weeks	Males, 20–28 years avg. 23.9 years, *n* = 18	TC = 4.71 HDL = 1.09 TAG = 1.2	1) TC ↓ 14.0% LDL ↓ 19.4% HDL ↓ 0.1% TAG ↓ 13.9% 2) TC ↑ 3.3% LDL ↓ 5.6% HDL ↑ 2.8% TAG ↑ 1.5%	1) *P* < 0.001 *P* < 0.001 NS *P* < 0.001 2) NS NS NS NS
Noakes and Clifton (1998)^32^	R, C, CRO	1) 15 E% TFA-free CO 2) 15 E% TFA CO	20 E% butter	3 weeks	Males and females, 41–66 years, *n* = 18	TC = 5.71 ± 0.23 LDL = 3.89 ± 0.18 HDL = 1.17 ± 0.08 TAG = 1.43 ± 0.11	1) TC ↓ 8.4% LDL ↓ 13.6% HDL ↑ 6.7% TAG ↓ 5.6% 2) TC ↓ 8.4% LDL ↓ 12.1% HDL ↓ 0.8% TAG ↓ 4.4%	1) *P* < 0.01 *P* < 0.01 NS NS 2) *P* < 0.01 *P* < 0.01 NS NS
Nydahl et al. (1994)^46^	DB, CRO	Dietary fat mainly from RO	1) Baseline habitual diet 2) SO diet	3 weeks	Males and females, avg. 29.2 ± 12.0 years, *n* = 101	TC = 4.79 ± 0.98 LDL = 3.24 ± 0.89 HDL = 1.24 ± 0.27 TAG = 1.02 ± 0.45	1) TC ↓ 4.0% LDL ↓ 4.9% HDL ↑ 1.6% TAG ↓ 3.9% 2) TC ↑ 0.4% LDL ↑ 2.0% HDL ↑ 2.4% TAG ↔ 0%	1) *P* < 0.001 *P* < 0.001 NS NS 2) NS NS NS NS
Palomäki et al. (2010)^33^	R, CRO	∼11–12 E% (35 mL/day) cold-pressed turnip LEAR oil	∼11–12 E% butter (37.5 g/day)	6–8 weeks	Males, 30–65 years, *n* = 37	TC = 5.15 ± 1.49 LDL = 3.33 ± 1.25 HDL = 1.15 ± 0.27 TAG = 2.19 ± 1.13	TC ↓ 8.3% LDL ↓ 10.4% HDL ↔ 0% TAG ↓ 5.9%	*P* < 0.001 *P* < 0.001 NS NS
Pedersen et al. (2000)^60^	R, DB, CRO	19 E% CO	1) 19 E% OO 2) 19 E% SO	3 weeks	Males, 20–28 years, *n* = 18	TC = 4.74 HDL = 1.10 TAG = 1.2	1) TC ↓ 11.6% LDL ↓ 19.9% HDL ↑ 1.0% TAG ↑ 15.1% 2) TC ↓ 1.9% LDL ↓ 8.5% HDL ↑ 8.8% TAG ↑ 1.3%	1) *P* < 0.001 *P* < 0.001 NS NS 2) NS NS NS NS
Sarkkinen et al. (1998)^22^	R, C, SB, P	RO and RO-based margarine, 33 E% fat	1) Baseline habitual diet 2) High-SFA diet (*n* = 36)	6 months	Males and females, 23–58 years, *n* = 41 (RO group)	TC = 6.54 ± 1.12 LDL = 4.55 ± 0.95 HDL = 1.35 ± 0.28 TAG = 1.55 ± 0.94	1) TC ↓ 3.7% LDL ↓ 6.6% HDL ↑ 2.2% TAG ↓ 9.7% 2) TC ↓ 3.5% LDL ↓ 3.0% HDL ↓ 10.4% TAG ↑ 2.9%	1) NS *P* < 0.01 NS NS 2) NS NS NS NS
Seppänen-Laakso et al. (1992)^47^	C, P	1) Avg. 7 E% RO (18 g/day) 2) Avg. 8 E% RO margarine (23 g/day)	Baseline habitual diet: avg. 7–8 E% butter	6 weeks	Males and females, avg. 43 years, *n* = 20/23 (RO group/RO margarine group)	TC = 6.32 ± 0.18 LDL = 4.39 ± 0.18 HDL = 1.47 ± 0.10 TAG = 0.99 ± 0.09	1) TC ↓ 3.0% LDL ↓ 6.4% HDL ↓ 2.0% TAG ↑ 16.2% 2) TC ↓ 1.3% LDL ↓ 3.3% HDL ↑ 0.7% TAG ↑ 14.1%	1) NS *P* < 0.05 NS *P* < 0.05 2) NS NS NS NS
Seppänen-Laakso et al. (1993)^48^	C, P	Avg. 6 E% RO (15% of total fat intake)	Baseline habitual diet: avg. 6 E% margarine	6 weeks	Males and females, 45.5 ± 2.0 years, *n* = 23 (RO group)	TC = 6.07 ± 0.14 LDL = 4.13 ± 0.14 HDL = 1.33 ± 0.05 TAG = 1.34 ± 0.12	TC ↓ 4.8% LDL ↓ 6.5% HDL ↑ 3.0% TAG ↓ 9.0%	NS NS NS NS
Södergren et al. (2001)^34^	R, C, SB, CRO	∼28 E% RO-based fat products	∼28 E% SFA products	4 weeks	Males and females, avg. 50 ± 8 years, *n* = 10 (RO group)	TC = 6.59 ± 1.02 LDL = 4.31 ± 0.94 HDL = 1.55 ± 0.40 TAG = 1.40 ± 0.61	TC ↓ 3.3% LDL ↓ 11.1% HDL ↑ 22.6% TAG ↓ 13.3	*P* < 0.001 *P* < 0.001 NS NS
Stricker et al. (2008)^49^	R, DB, P	2 tablespoons/day CO	Baseline habitual diet	8 weeks	Males and females, avg. 66.8 ± 8.1 years, *n* = 20 (CO group)	TC = 4.73 ± 0.9 LDL = 2.74 ± 0.73 HDL = 1.44 ± 0.32 TAG = 1.44 ± 0.55	TC ↓ 6.6% LDL ↓ 11.3% HDL ↑ 1.4% TAG ↓ 2.3%	*P* < 0.05 *P* < 0.01 NS NS
Sundram et al. (1995)^40^	R, C, DB, CRO	∼20 E% CO	∼20 E% palm olein	4 weeks	Males, 19–24 years, *n* = 23	TC = 4.46 ± 0.64 LDL = 2.64 ± 0.51 HDL = 1.18 ± 0.26 TAG = 0.94 ± 0.36	TC ↓ 2.2% LDL ↓ 4.5% HDL ↔ 0% TAG ↑ 9.6%	NS NS NS NS
Uusitupa et al. (1994)^35^	R, C, CRO	LEAR oil-based margarine as main fat source, 40 E% fat, 10 E% SFA	High-SFA diet, 40 E% fat, 20 E% SFA, butter main fat source	3 weeks	Females, avg. 23 ± 1.6 years, *n* = 10	TC = 5.21 ± 0.92 TAG = 0.76 ± 0.21	TC ↓ 21.7% LDL ↓ 29.4% HDL ↓ 1.9% TAG ↑ 7.7%	*P* < 0.001 *P* < 0.001 NS NS
Valsta et al. (1992)^36^	DB, CRO	63% of MUFA from LEAR oil (∼10 E%)	1) Baseline (2 wk): 18.9 E% SFA 2) SO diet: 87% of PUFA from SO (∼12 E%)	3 weeks	Males and females, 18–65 years, *n* = 59	TC = 5.35 ± 0.98 LDL = 3.17 ± 0.82 HDL = 1.33 ± 0.28 TAG = 0.88 ± 0.37	1) TC ↓ 15.5% LDL ↓ 24.0% HDL ↑ 0.8% TAG ↓ 3.4% 2) TC ↓ 2.2% LDL ↓ 6.6% HDL ↔ 0% TAG ↑ 7.6%	1) *P* < 0.001 *P* < 0.05 NS Not reported 2) *P* < 0.01 *P* < 0.01 NS *P* < 0.05
Vega-López et al. (2006)^41^	R, C, DB, CRO	∼13 E% CO (2/3 of total fat intake)	∼13 E% palm oil (2/3 of total fat intake)	35 days (5 weeks)	Males and females, ≥50 years, *n* = 15	TC = 6.54 LDL = 4.58 HDL = 1.40 TAG = 1.25	TC ↓ 12.5% LDL ↓ 14.2% HDL ↓ 3.6% TAG ↔ 0%	*P* < 0.05 *P* < 0.05 NS NS
Wardlaw et al. (1990)^54^	R, C, SB, P	>31.2 E% CO, 39 E% fat	Baseline (3 wk, *n* = 32): Western diet, avg. 39% fat (mainly butter) and 15% SFA	8 weeks	Males, 21–47 years, *n* = 16 (CO group)	TC = 5.36 ± 0.15 LDL = 3.64 ± 0.15 HDL = 1.12 ± 0.05 TAG = 1.47 ± 0.16	TC ↓ 8.8% LDL ↓ 11.1 HDL ↑ 0.9% TAG ↓ 7.9%	*P* < 0.01 *P* < 0.01 NS NS

Magnitude of effect (%)^a^ of TC/LDL/HDL/TG = (canola oil treatment-control diet)/control diet ^*^100%.

*Abbreviations and symbols*: ↓, decrease; ↑, increase; ↔, no change; C, control group; CO, canola oil; CRO, crossover; DB, double blind; E%, percentage of total energy intake; HDL, high-density lipoprotein; LDL, low-density lipoprotein; LEAR, low-erucic acid rapeseed; MUFA, monounsaturated fatty acids; NS, non-significant; OO, olive oil; P, parallel; PUFA, polyunsaturated fatty acids; R, randomized; RO, rapeseed oil; SB, single blind; SFA, saturated fatty acids; SO, sunflower oil; TAG, triacylglycerol; TC, total cholesterol; TFA, *trans* fatty acid.

**Table 2:** Summary of studies investigating biomarkers of antioxidants, hemostasis, lipid peroxidation, and inflammation with different types of diets

Biomarker	Control diet	Effect of canola/LEAR oil	Reference
Antioxidants			
α-tocopherol	Baseline SFA diet	Increase	Gustafsson et al. (1994)^26^
	SO diet	No change	Gustafsson et al. (1994)^26^
	SFA diet	No change	Södergren et al. (2001)^34^
γ-tocopherol	Baseline SFA diet	No change	Gustafsson et al. (1994)^26^
	SO diet	Increase	Gustafsson et al. (1994)^26^
	SFA diet	Increase	Södergren et al. (2001)^34^
Hemostasis			
Platelet aggregation	Safflower diet	No change	Kwon et al. (1991)^65^
	Baseline SFA diet	Decrease	Kwon et al. (1991)^65^
Collagen-induced platelet aggregation	High-SFA diet/SO diet	No change	McDonald et al. (1989)^28^
Thromboxane B_2_	SO, OO, and FO/SO and OO/soybean oil diets	No change	Chan et al. (1993)^68^
	Safflower oil diet	No change	Kwon et al. (1991)^65^
	High-SFA diet	No change	McDonald et al. (1989)^28^
11-dehydro-thromboxane B_2_	SFA diet	No change	Södergren et al. (2001)^34^
6-keto prostaglandin_1α_	SO, OO, and FO/SO and OO/soybean oil diets	No change	Chan et al. (1993)^68^
	High-SFA diet	Increase	McDonald et al. (1989)^28^
Factor VII coagulant activity (FVIIa)	Baseline habitual diet	Decrease	Iggman et al. (2011)^38^
	Baseline SFA diet	No change	Junker et al. (2001)^51^
Coagulation factor VIIc (FVllc)	Baseline habitual diet	No change	Junker et al. (2001)^51^
Coagulation factor XII (FXIIc)	Baseline habitual diet	No change	Junker et al. (2001)^51^
Coagulation factor XIIa (FXIIa)	Baseline habitual diet	No change	Junker et al. (2001)^51^
Plasminogen activator inhibitor-1	Dairy fat diet	No change	Iggman et al. (2011)^38^
Fibrinogen	Dairy fat diet	No change	Iggman et al. (2011)^38^
	Baseline SFA diet	No change	Junker et al. (2001)^51^
Pathromtin SL (FIXc)	Baseline SFA diet	No change	Junker et al. (2001)^51^
Pathromtin SL (FXc)	Baseline SFA diet	No change	Junker et al. (2001)^51^
Pathromtin SL (Fllc)	Baseline SFA diet	No change	Junker et al. (2001)^51^
Pathromtin fragment (F1+2)	Baseline SFA diet	No change	Junker et al. (2001)^51^
Procoagulant activity	Control diet	No change	Mendez et al. (1996)^69^
ATP secretion	Baseline SFA diet	Decrease	Kwon et al. (1991)^65^
Bleeding time	SO, OO, and FO/SO and OO/soybean oil diets	No change	Chan et al. (1993)^68^
	Mixed-fat SFA diet	Increase	McDonald et al. (1989)^28^
Lipid peroxidation			
8-iso prostaglandin_2α_	SFA diet	No change	Södergren et al. (2001)^34^
Conjugated dienes	Palm oil	Decrease	Mutalib et al. (1995)^43^
Thiobarbituric acid reactive substances	Palm oil	No change	Mutalib et al. (1995)^43^
Glutathione peroxidase activity	Palm oil	Decrease	Mutalib et al. (1995)^43^
Erythrocyte superoxide dismutase activity	Palm oil	Decrease	Mutalib et al. (1995)^43^
Hydroperoxides	SFA diet	No change	Södergren et al. (2001)^34^
Malondialdehyde	SFA diet	No change	Södergren et al. (2001)^34^
Inflammation			
C-reactive protein	High-oleic CO and FO/high-SFA diets	No change	Gillingham et al. (2011)^25^
	Baseline SFA diet	No change	Junker et al. (2001)^51^
Interleukin-6	High-oleic CO and FO/high-SFA diets	No change	Gillingham et al. (2011)^25^
	Cream/OO/potato/All-Bran diets	No change	Manning et al. (2008)^70^
sVCAM-1	High-oleic CO and FO/high-SFA diets	No change	Gillingham et al. (2011)^25^
sICAM-1	High-oleic CO and FO/high-SFA diets	No change	Gillingham et al. (2011)^25^
Soluble E-selectin	High-oleic CO and FO/high-SFA diets	No change	Gillingham et al. (2011)^25^
P-selectin	Baseline SFA diet	No change	Junker et al. (2001)^51^
L-selectin	Baseline SFA diet	No change	Junker et al. (2001)^51^
Tumor necrosis factor	Diet without CO, arginine, and certain trace elements	Improved	Mendez et al. (1996)^69^
Tumor necrosis factor-α	Cream/OO/potato/All-Bran diets	No change	Manning et al. (2008)^70^
Interleukin-8	Cream/OO/potato/All-Bran diets	No change	Manning et al. (2008)^70^
Prostaglandin E_2_	Diet without CO, arginine, and certain trace elements	Increase	Mendez et al. (1996)^69^
Cortisol	Cream/OO/potato/All-Bran diets	No change	Manning et al. (2008)^70^
Neutrophil oxidative burst	Diet without CO, arginine, and certain trace elements	No change	Mendez et al. (1996)^69^
Lymphocyte proliferation	Diet without CO, arginine, and certain trace elements	No change	Mendez et al. (1996)^69^

*Abbreviations*: CO, canola oil; FO, flaxseed oil; OO, olive oil; SFA, saturated fatty acids; sICAM-1, soluble intercellular adhesion molecule-1; SO, sunflower oil;VCAM-1, soluble vascular cell adhesion molecule.

## Results

### Canola oil and regulation of circulating lipid profiles

Thirty-one studies examined the effects of canola oil-based diets on circulating lipid subclass levels in clinical intervention trials. The available studies, summarized in Table 1, demonstrate heterogeneity in terms of the study population chosen, including gender, age range, blood lipid levels, and health status. Two studies evaluated the longer-term effects of canola oil on circulating lipid levels.^21^,^22^ Gulesserian et al.^21^ compared baseline and endpoint serum cholesterol levels after 5 months in which children and adolescents with familial hypercholesterolemia received dietary counselling and were instructed to replace as many visible fats as possible by canola oil. Data revealed significant reductions of TC, LDL-C, and triacylglycerol (TAG) levels in the participants. It should be noted, however, that participants were also instructed to eat high quantities of fruits and vegetables and include a weekly meal of fish in their diet. Thus, it cannot be concluded that canola oil alone was responsible for the favorable changes observed. Sarkkinen et al.^22^ conducted a 6-month dietary intervention study in hypercholesterolemic adults and found that LDL-C levels were lowered (3.7%) from baseline in the rapeseed group; however, no significant differences were found in serum TC, LDL-C, HDL-C, and TAG levels between canola oil-based diets and diets with oils higher in SFAs. A study such as this that extends over several months may be associated with compliance problems and seasonality effects, which could explain why no differences were observed in endpoint cholesterol levels between the treatment and control groups. 23

In 29 studies, the effects of canola oil on circulatory cholesterol levels were examined with intervention periods ranging from 2.5 to 8 weeks, and an effect was seen in most of these short-term studies. Since the lipid-modulating effects of canola oil may be dependent on the types of fatty acids that are replaced by the oil, the effect of canola oil on blood lipid levels are discussed separately for replacement of SFAs or other vegetable oils.

These studies indicate that canola oil has the potential to help consumers meet dietary fat recommendations and can be included in a diet designed to regulate serum cholesterol levels in order to improve the condition of hypercholesterolemia.^24^

### Effects of canola oil compared to saturated fatty acids on blood lipid profiles

SFAs are found in animal fat and dairy products as well as certain types of vegetable oils, including coconut and palm oils. Typical Western diets are high in SFAs. A high amount of dietary SFA may increase TC and LDL-C levels, thus increasing the risk of developing cardiovascular disease.^16^ Efforts to substitute SFAs have typically relied on replacement of animal fat and dairy products with liquid vegetable oils high in MUFAs and PUFAs.^14^ A total of 27 studies, of the 31 studies summarized in Table 1, specifically examined the blood lipid-modulating effects of diets containing canola oil compared with high-SFA or Western diets; these 27 studies are discussed in more detail below.

#### Total cholesterol levels

Five studies compared canola oil-based diets with typical Western diets,^25^–^29^ providing evidence that canola oil is able to reduce TC levels by an average of 12.2% (range, 6.7–20.1%). Diets based on canola oil showed positive effects on TC levels regardless of the type of canola oil used compared to a typical Western diet. For example, Gillingham et al.^25^ selected high-oleic canola oil (>70% oleic acid) rather than conventional canola oil (>60%) for their canola-based diet and found a 6.7% reduction of TC levels compared with typical Western diets.

Seven studies compared canola oil-based diets with those high in SFAs.^30^–^36^ The high-SFA diets in these studies contained >12% of energy from SFAs and >30% of energy from total fat, mainly in the form of butter, margarine, mayonnaise, and other high-SFA foods. Five of the seven studies examined the effects of margarines or mayonnaises made with canola oil on TC levels^30^–^35^ and all showed a lowering of TC levels, in a range from 3.3% to 21.7%, compared with a high-SFA-treated group. Palomäki et al.^33^ and Valsta et al.^36^ fed their subjects diets containing canola oil or a high-SFA alternative rather than incorporating the oil into food products; their results demonstrated significant lowering of TC levels with the canola oil, at 8.3% and 15.5%, respectively, compared with the high-SFA alternative. Results from all seven studies consistently showed that replacing higher SFA dietswith canola oil decreased TC levels.

Baudet and Jacotot,^37^ Iggman et al.,^38^ and Karvonen et al.^39^ compared dietary canola oil with various high-SFA dairy products. Substituting canola oil for dairy fat caused TC levels to decrease by 18.4%,^37^ 16.1%,^38^ and 5.1%,^39^ respectively, in these three studies. Current food technology allows substitution of SFA with canola oil in dairy manufacturing, which may offer new options for regulating blood cholesterol levels and, thereby, reduce the risk of cardiovascular disease.

SFAs are not only found in animal fat, they are also derived from vegetable oils such as coconut and palm oils. Some commercial food manufacturers have selected high-SFA vegetable oils as alternatives to animal fat because of taste. When comparing canola oil-based to palm oil-based diets, Sundram et al.^40^ found a 2.2% reduction in TC and Vega-López et al.^41^ observed a 12.5% decrease in TC levels with the canola oil-based diets. McKenney et al.^42^ designed two studies in which hypercholesterolemic participants consumed cookies made with either canola or coconut oil. Levels of TC were reduced by an average of 8.7% in groups consuming the canola oil-containing cookies. Not only can high-SFA vegetable oils replace animal SFA, hydrogenated vegetable oils are also considered substitutes for SFAs.^43^,^44^ Mutalib et al.^43^ found that hydrogenated rapeseed oil decreased TC and LDL and increased HDL levels compared to palm oil. Since errors were found in this study's abstract, the present analysis was based on the data found solely within the tables of the article.

Although several studies defined their baseline diets as high-SFA (>10% SFAs) or Western diets (>30% total fat), some investigations failed to evaluate the participants' diets before intervention.^45^–^51^ These studies, therefore, refer to participants' consumption patterns as their “habitual diets.” A few reports examined the effects of canola oil interventions on TC levels compared to habitual diets. Miettinen and Vanhanen,^45^ Nydahl et al.,^46^ Stricker et al.,^49^ Corboy et al.,^50^ and Junker et al.^51^ showed that canola oil-based diets significantly lowered TC levels. However, two studies from Seppänen-Laakso et al.^47^,^48^ compared canola oil-based margarine with butter and margarine in participants' habitual diet and failed to show statistically significant differences between the two diets.

Overall, clinical studies have shown that compared with high-SFA or typical Western diets, canola oil-based diets can reduce TC concentrations in healthy or hypercholesterolemic individuals.

#### Low-density lipoprotein cholesterol levels

In general, SFA consumption is associated with elevations in LDL-C. Replacing SFAs with MUFAs can reduce circulatory cholesterol levels, especially LDL-C levels, and decrease atherosclerosis and CHD risk.^52^ Replacing 5% of SFAs with oleic acid can reduce CHD risk by as much as 20–40% through LDL-C level suppression.^13^,^53^ From the short-term interventional clinical studies on LDL-C, reviewed and summarized in Table 1, it is clear that the impact of canola oil-based diets versus high-SFA or Western diets on reducing LDL-C is comparable to its TC-lowering impact. Five studies compared canola oil-based diets with a typical Western diet.^25^–^28^,^54^ These studies found that canola oil reduced LDL-C levels by an average of 17%, with LDL-C reductions ranging from 11.1% to 25.2%. This variability in the reduction of LDL-C levels may be explained, in part, by the sample size of the experimental groups, since the greatest reduction in LDL-C levels was observed in a group of only four university students.^28^ Higher baseline LDL-C levels were also associated with greater reductions in LDL-C concentrations during the intervention period. For instance, in four studies, participants' baseline LDL-C levels were at 4.85,^26^ 3.93,^27^ 3.7,^25^ and 3.6^54^ mmol/L, and they were reduced by 19.6%,^26^ 17.0%,^27^ 12.2%,^25^ and 11.1%,^54^ respectively, after the canola oil-based diets were followed. Thus, the canola oil-based diets elicited consistent effects on LDL-C lowering compared to the Western diet and were strongly associated with the participants' baseline levels.

Several studies were designed to specifically compare canola oil-based diets with high-SFA diets.^30^–^36^ These studies all showed significant reductions in LDL-C levels after canola oil-based diets were consumed, with an average reduction of 16.2% (range, 10–29.4%). In particular, Valsta et al.^36^ reported a 24% reduction in LDL-C following a canola oil-based diet compared with a baseline high-SFA diet. Uusitupa et al.^35^ designed a high-fat diet using either high SFA or high canola oil. The LDL-C level was 29.4% lower in the high-canola-oil diet compared to the high-SFA diet. Valsta et al.^36^ and Uusitupa et al.^35^ suggested that substitution of SFAs with canola oil strongly influenced LDL metabolism.

In other work, Baudet and Jacotot,^37^ Iggman et al.,^38^ and Karvonen et al.^39^ indicated that substitution of dairy fats with canola oil significantly reduced LDL-C levels by 24.9%, 19.6%, and 6.4%, respectively. In the study by Karvonen et al.,^39^ participants consumed either canola oil-based cheese or milk fat-based cheese without changing their habitual diet. In the other two studies,^37^,^38^ dairy fats in the diet were replaced with canola oil.

Similarly, Sundram et al.,^40^ Vega-López et al.,^41^ and McKenney et al.^42^ analyzed levels of LDL-C in participants consuming either canola oil or high-SFA vegetable oils, such as palm or coconut oil. Results showed that canola oil-based diets significantly lowered LDL-C levels by average of 8.9%. It is important to realize that, in comparison with palm oil-based diets, the canola oil-based diet lowered LDL-C concentrations by 4.5% in the study by Sundram et al.^40^ and by 14.2% in the study by Vega-López et al.^41^ This disparity may relate to the participants' demographic characteristics and to the total fat content in the experimental diets. For instance, Sundram et al.^40^ selected healthy young males with an average baseline LDL-C level of 2.64 mmol/L after 2 weeks on a high-fat diet. In contrast, Vega-López et al.^41^ examined males and females (>50 years) with high baseline LDL-C levels (average, 4.5 mmol/L).

Effects of canola oil on LDL-C levels compared with subjects' baseline habitual diets have been inconsistent and, again, this is likely because of the different treatment designs.^45^–^51^ For example, Nydahl et al.,^46^ Seppänen-Laakso et al.,^47^ Stricker et al.,^49^ Corboy et al.,^50^ and Junker et al.^51^ showed LDL-C reductions in association with canola oil-based diets compared with baseline habitual diets. Similarly, Miettinen and Vanhanen^45^ showed reductions in LDL-C when traditional mayonnaise was replaced with canola oil-based mayonnaise. Nevertheless, Seppänen-Laakso et al.^47^,^48^ failed to identify any reduction in LDL-C levels when traditional margarine was isocalorically replaced with either canola oil or margarine made with canola oil. Hence, based on participants' lifestyle, food preferences, and other background factors, it is difficult to summarize the effects of canola oil-based diets against habitual diets.

#### High-density lipoprotein cholesterol levels

High-density lipoproteins (HDL-C) assist in the elimination of excess cholesterol. Higher HDL-C levels have been associated with lower risk of CHD, while low HDL-C has been identified as an independent risk factor associated with CHD.^55^–^58^ Therefore, increasing HDL-C is important for preventing and treating CHD.

The same 27 short-term studies that evaluated the blood lipid modulating effects of canola oil-based diets versus high-SFA/Western diets also examined the effects on HDL-C levels. In 22 of the 27 studies, regardless of whether the assessment was between canola oil-based and high-SFA/Western or habitual diets, statistically significant diet-related differences in HDL-C levels were not observed. In contrast, Gustafsson et al.,^26^ Lichtenstein et al.,^27^ McDonald et al.,^28^ and Hodson et al.^30^ reported reductions in HDL-C concentrations following consumption of canola oil-based diets compared with high-SFA/Western diets. However, the TC/HDL-C ratio did not change significantly.^26^,^30^ Miettinen and Vanhanen^45^ found an increase in HDL-C after a canola oil mayonnaise-based diet compared to a baseline habitual diet. Hodson et al.^30^ indicated that if total and saturated fat intake were decreased while PUFA or MUFA intakes were increased, HDL-C levels were reduced. However, it should be noted that in most of the studies reviewed, canola oil-based diets had no observed effects on HDL-C levels compared with high-SFA/Western diets.

#### Triacylglycerol levels

TAGs exist as the most common form of fat in the human gastrointestinal tract and represent the main constituents of vegetable oils and animal fats. Excessive intakes of dietary fats, especially SFAs, result in increased circulating TAG concentrations. High-carbohydrate diets, particularly when foods high in simple sugars are consumed, also contribute significantly to high circulatory TAG levels. Therefore, instead of replacing SFAs with carbohydrates, it is commonly believed that limiting total fat intake and choosing MUFAs and PUFAs instead of SFAs represent the most effective strategies for reducing circulating TAG levels. While 21 of 27 short-term studies were unable to show statistically significant changes in TAG levels between canola oil- and high-SFA/Western-type diets, four studies did show a reduction on a canola oil-based diet and one showed an increase. Iggman et al.^38^ concluded that replacing dairy fat with canola oil within a high-fat diet improved TAG levels in participants. Another three studies^26^,^30^,^45^ supported the conclusion of Iggman et al.,^38^ showing a reduction in TAG levels by an average of 15.8% following consumption of a canola oil-based diet. However, two studies^19^,^51^ showed an increase in TAG levels in participants on a canola oil diet compared with the high-SFA baseline diet.

### Effects of canola oil versus other MUFA- and PUFA-rich vegetable oils on blood lipid profiles

As summarized in Table 1, five studies compared canola^27^,^36^,^46^,^59^,^60^ or high-oleic canola^25^ oil to other high-MUFA or high-PUFA vegetable oils, including a blend of flaxseed and high-oleic canola oils (50/50%) or corn, olive, or sunflower oils.

#### Total cholesterol levels

Six studies examined consumption of canola oil versus high-PUFA oils. In four of these six studies,^36^,^46^,^59^,^60^ sunflower oil was chosen as one of the control oils. No significant differences were observed between canola and sunflower oils on the extent of reduction of TC concentration compared to baseline, resulting in the conclusion that sunflower oil can improve TC concentration as well as canola oil. Three studies^27^,^59^,^60^ selected olive oil as the control against canola oil. Results showed TC concentration reductions of 12.2%,^27^ 14.0%,^59^ and 11.6%^60^ after consumption of canola oil compared to olive oil. Gillingham et al.^25^ compared high-oleic canola oil with a blend of flaxseed and high-oleic canola oils. Greater improvements in TC concentrations were seen with the oil blend treatment, which was higher in ALA, than with the high-oleic canola oil treatment alone. Lichtenstein et al.^27^ showed that consumption of canola oil reduced TC concentration by 5.4%, on average, compared to corn oil.

#### Low-density lipoprotein cholesterol levels

Interestingly, the reductions in LDL-C levels achieved with canola oil were similar when compared with corn and sunflower oils or an oil blend of flaxseed and high-oleic canola oils. However, three studies^27^,^59^,^60^ showed a higher reduction of LDL-C concentrations with canola oil-based diets compared with diets using olive oil. Truswell and Choudhury^61^ indicated that oils high in MUFAs do not have the same effect on plasma cholesterol because, in their study, olive oil was associated with higher TC and LDL-C levels compared with canola or high-oleic sunflower oils. This finding suggests canola oil may have more beneficial effects on LDL-C regulation than olive oil.

#### High-density lipoprotein cholesterol levels

According to research by Hodson et al.,^30^ when MUFA intake is increased while total and SFA intakes decrease, the HDL-C level is reduced to a lesser extent than when PUFA intake is increased. Gillingham et al.^25^ reported that a diet with high-oleic canola oil led to higher HDL-C concentrations compared to a diet with a blend of flaxseed and high-oleic canola oils. However, no significant differences in HDL-C concentration were observed between canola oil-based and other vegetable oil-based diets.

#### Triacylglycerol levels

With one exception, none of the studies^25^,^27^,^46^,^59^,^60^ reviewed showed differences in TAG levels with conventional and high-oleic canola oils versus other vegetable oils, including a flaxseed/high-oleic canola oil blend and corn, olive, and sunflower oils. In the exception, Valsta et al.^36^ reported that levels of TAG increased by 7.6% in a canola oil-based diet compared with a sunflower oil-based diet. Despite this, the TAG levels after the canola oil treatment (0.85 mmol/L) were still within normal ranges. In general, canola oil-based diets appear to have neutral effects on TAG levels for participants with normal levels of TAG at baseline.

The findings regarding the effect of canola oil on TAG indicate that important evidence exists to support a beneficial role of canola oil in CHD by regulating circulating lipid profiles.

From the results of the existing 31 studies, it can be concluded that replacing SFAs with canola oil in Western-type or high-fat diets is beneficial for decreasing TC, LDL, and maintaining normal TAG levels. Despite canola oil showing a protective effect in some studies, the results of studies comparing canola oil with other vegetable oils (e.g., MUFA- or PUFA-rich) are less consistent. For example, not only did canola oil reduce baseline TC and LDL levels, sunflower oil also appeared to be advantageous in regulating lipid profiles, while olive oil appeared less efficient compared with sunflower oil or canola oil. However, the number of such studies appears to be too small to allow conclusions to be drawn, particularly in light of differences in study design, sample size, or baseline status.

### Effects of canola oil on susceptibility of low-density lipoprotein to oxidation and lipid peroxidation

Many physiological factors beyond lipid levels are associated with risk of CHD, including biomarkers of platelet formation, coagulation, inflammation, and lipid peroxidation. Oxidized LDL could be a risk factor for CHD by promoting atherogenesis^50^ and butylated hydroxytoluence (BHT).^50^ Several factors are associated with the susceptibility of LDL-C to oxidative modification, including dietary fatty acid composition. For example, the dietary PUFA content in lipoproteins tends to increase the sensitivity of those particles to oxidative damage, while dietary MUFA content results in LDL-C particles that are less prone to oxidation.^50^ Furthermore, the quantity and density of LDL-C particles may be important in terms of their susceptibility to oxidation.^59^ Due to the above considerations, many studies have analyzed the effects of different dietary oils on their potential to oxidize LDL-C.

Södergren et al.^34^ examined the effects of a canola oil-based diet compared to an SFA-based diet on biomarkers of lipid peroxidation. Because of the presence of easily oxidized PUFA in canola oil, it could be hypothesized that the degree of lipid peroxidation would increase when following the canola oil diet compared to the SFA diet. However, no significant differences in the levels of the tested biomarkers were found.

Corboy et al.^50^ examined the effect of replacing dietary fat with LEAR oil in a muesli diet by measuring LDL susceptibility to copper-catalyzed oxidation in vitro. Results showed that 18 subjects who consumed the rapeseed oil/muesli diet for 4 weeks showed reduced susceptibility to in vitro LDL oxidation compared to the level of susceptibility with their baseline normal diet.

Turpeinen et al.^62^ designed two studies to examine the effects of dietary oil on lipid peroxidation. In the first study, 59 healthy subjects (30 female, 29 male) were given a milk fat-based (SFA) diet for 14 days, after which 30 subjects consumed for 24 days a sunflower oil-based (PUFA) diet, then for another 24 days a LEAR oil-based (MUFA) diet; the other 29 subjects ingested the vegetable oils in reverse order. In the second study, 13 healthy male subjects were divided into two groups, with 6 subjects consuming a LEAR oil-based diet (50 g/day) and 7 subjects consuming a sunflower oil-based diet (50 g/day) for 6 weeks in a parallel manner. In the first study, LEAR oil diet consumption was consistent with a lower plasma concentration of malondialdehyde and glutathione compared with the baseline milk fat diet. However, the sunflower oil diet failed to show the same effect. In the second study, the LEAR oil-based diet resulted in increased lag time and time to maximum oxidation and decreased oxidation velocity, while results from the sunflower oil-based diet were opposite. While both the LEAR oil-based and sunflower oil-based diets showed changes in the LDL fraction in thiobarbituric acid reactive substances (TBARS) and lipid hydroperoxide compared to baseline, the sunflower oil-based diet showed a significant decrease of TBARS and lipid hydroperoxide compared to the LEAR oil-based diet.

Palomäki et al.^33^ examined the effects of cold-pressed LEAR oil on LDL-C oxidation for periods of 6–8 weeks in 37 men with metabolic syndrome. Results showed that the level of oxidized LDL-C was 16% lower after butter was replaced with LEAR oil. This study indicated that LEAR oil was beneficial in lowering LDL-C oxidation. Cold pressing typically results in retention of more of the antioxidant-like molecules in vegetable oils compared to conventional processing. Since the cold-pressed oil was not specifically compared to conventionally-processed LEAR oil, it is not known if the above benefits would be seen with conventional LEAR oil.

Nielsen et al.^59^ measured the effects of different unsaturated fatty acid diets on oxidation of fasting and postprandial lipoproteins in a double-blind, randomized crossover study. Healthy men consumed diets with canola, olive, or sunflower oil for 3 weeks each. No differences were found in the lag time of LDL-C oxidation across the three diets; however, the sunflower oil-based diet increased susceptibility to oxidation in fasting and postprandial stages compared with the canola or olive oil-rich diets. Kratz et al.^63^ showed that the extent of LDL-C oxidation aligned with the tested dietary patterns as follows: sunflower oil > canola oil > olive oil. Schwab et al.^64^ reported no differences in susceptibility of LDL-C to oxidation after consumption of reduced-fat diets enriched with either animal fat or vegetable oils, including canola, corn, olive, and rice bran oils. Mutalib et al.^43^ indicated that after an 8-week feeding period, healthy subjects in the palm oil treatment group showed reduced indices of lipid peroxidation. Here, the hydrogenated rapeseed oil treatment group decreased (*P* < 0.05) plasma conjugated dienes and erythrocyte superoxide dismutase activity compared with the palm oil treatment group, but no differences were observed in TBARS levels or glutathione peroxidise activity.

In summary, although studies examining the effects of conventional canola oil-based diets on lipid peroxidation and susceptibility of LDL-C to oxidation failed to show significant effects, some studies suggested that replacing SFA with vegetable oil may affect lipoprotein susceptibility of LDL to oxidation in vitro,^50^,^62^ but there was considerable disparity in the in vivo lipid peroxidation data. Other work has shown that oleic acid reduces LDL-C oxidation.^64^ These findings may indicate a potential role for canola oil, which contains 61% oleic acid, in the regulation of LDL-C oxidizability.

### Effects of canola oil on biomarkers of hemostasis and inflammation

Epidemiological studies in Greenland Inuit cohorts^65^–^67^ have shown that a lower incidence of CHD mortality is not only associated with lower plasma TC and TAG levels, but also with decreased platelet aggregation in vitro as well as lower thromboxane A_2_ (TXA_2_) synthesis rates. TXA_2_ is a prothrombotic agent contributing to platelet activation and aggregation and is the principal metabolite of arachidonic acid, an omega-6 fatty acid. In contrast, the omega-3 fatty acid eicosapentaenoic acid can be converted to TXA_3,_ having lesser platelet aggregatory activity relative to TXA_2_. Several studies have investigated the effects of a canola oil-based diet on biomarkers of antioxidant status, platelet aggregation, and inflammation in the context of CHD risk (Table 2). Kwon et al.^65^ examined the effects of diets high in MUFA (canola oil) or PUFA (safflower oil) on platelet aggregation and formation of thromboxane B_2_ (TXB_2_), the prothrombotic stable metabolite of TXA_2_. After 3 weeks, the canola and safflower oil groups both showed decreases in platelet aggregation compared with the high-SFA baseline diet, but values returned to baseline levels at week 8. Only canola oil influenced platelet function by lowering ATP secretion at week 8. No significant differences in TXB_2_ concentrations were observed between the dietary intervention groups. Taken together, canola and safflower oil-based diets both resulted in temporarily reduced platelet aggregation compared to an SFA-based diet. However, the canola oil-based diet resulted in longer beneficial effects on platelet function than the safflower oil-based diet.

McDonald et al.^28^ observed the effects of the following diets on hemostasis: a high-SFA, mixed-fat diet; high-MUFA, canola oil-based diet; and high-PUFA, sunflower oil-based diet. Compared with the high-SFA, mixed-fat diet, only the canola oil-based diet significantly increased mean bleeding time and 6-keto prostaglandin1α production. 6-Keto prostaglandin1α is the stable metabolite of prostacyclin, which is known to induce vasodilation and inhibit platelet aggregation. TXB_2_ and collagen-induced platelet aggregation did not change with the canola oil-based diet, but were lowered with the sunflower oil-based diet compared to the mixed-fat diet. Overall, canola oil and sunflower oil showed similar antithrombotic effects when compared to a high-SFA diet. Södergren et al.^34^ also measured TXB_2_ concentrations and did not find significant differences between a canola oil-based and an SFA-based diet. In addition, Chan et al.^68^ investigated the effects of different dietary fat patterns and found no differences in bleeding time or TXB_2_ production during bleeding time. However, TXB_2_ production tended (*P* > 0.05) to be lower and the 6-keto prostaglandin1α to TXB_2_ ratio tended (*P* > 0.05) to be higher in individuals following the canola oil-based diet and a diet containing a blend of sunflower, olive, and flax oils than in individuals following a soybean oil-based diet or a diet containing a mixture of sunflower and olive oils.

Other studies have examined the influence of dietary oils on various immune functions and coagulation. Iggman et al.^38^ investigated the effects of a canola oil-based diet on coagulation compared with a diet based on dairy fat. After the feeding period, the canola oil-based diet showed a reduction in factor VII coagulant activity, suggesting that a canola oil-based diet may be preferable to an SFA-based diet in subjects with elevated coagulation factors. Seppänen-Laakso et al.^47^ found decreased fibrinogen levels after replacing SFA with canola oil in individuals with elevated baseline fibrinogen levels, suggesting that canola oil might favorably affect hemostasis by reducing fibrinogen. Mendez et al.^69^ examined the effects of an experimental diet containing canola oil, arginine, and select trace elements on immune function in critically injured patients, compared to a control diet without these dietary components. The leukocyte response from patients on the experimental diet normalized after 6 days, while the leukocyte response in patients on the control diet remained depressed. The experimental diet contained 40% of fat as canola oil; however, it was difficult to deduce the individual contribution of canola oil in the experimental diet to the observed effects. Manning et al.^70^ probed the acute effects of potato starch meals with or without added cream, olive oil, and canola oil on inflammatory markers in obese and lean women. Postprandial concentrations of plasma interleukin-6, interleukin-8, and tumor necrosis factor-α did not significantly differ among test meals. Likewise, Gillingham et al.^25^ failed to observe differences in plasma inflammatory markers between the experimental diets containing high-oleic canola oil, a blend of high-oleic canola and flaxseed oils, and a blend of oils typical of the Western diet. Also, Junker et al.^51^ failed to find changes in most of the coagulation factors between the SFA baseline diet and after the LEAR oil treatment diet; however, a reduction of coagulation factors (including FVIIc, FXIIc, FXIIa, and FXc) was found in both the olive oil and sunflower oil treatment diets compared to the baseline SFA diet.

Södergren et al.^34^ examined the effects of a canola oil-based diet compared to an SFA-based diet on biomarkers of antioxidative capacity. Levels of γ-tocopherol were higher following the canola oil-based diet compared to the SFA-based diet.^34^ Because of its antioxidant activity, the increased levels of γ-tocopherol may have protected the PUFA in the canola oil diet from oxidation. No differences in α-tocopherol were observed between the two diets, resulting in a decreased α- to γ-tocopherol ratio with the canola oil-based diet compared to the SFA-based diet. The relevance of this finding is demonstrated by other studies that associated low γ-tocopherol concentrations and a high α- to γ-tocopherol ratio with CHD incidence.^71^,^72^ Gustafsson et al.^26^ also examined tocopherol concentrations following consumption of a canola oil-based diet, but they used a sunflower oil-based diet for comparison. Both the canola and the sunflower oil diets increased α-tocopherol levels compared to the high-SFA baseline diet, which underscored that these oils are potentially beneficial for reducing the risk of ischemic heart disease in people with low plasma concentrations of vitamin E.^26^,^73^,^74^ Concentrations of γ-tocopherol decreased after the sunflower oil-based diet but did not change following the canola oil-based diet. This resulted in a substantial increase in the ratio of α- to γ-tocopherol following the sunflower oil diet while the canola oil diet showed more favorable results and only slightly increased the α- to γ-tocopherol ratio. In summary, canola oil may potentially promote immune and cardiovascular health through its antithrombic and antioxidative effects.

### Effects of canola oil on energy metabolism in in vitro and in vivo studies

Not all dietary fats undergo equivalent partitioning for energy expenditure and some may exhibit satiety-promoting actions.^75^ To help individuals maintain normal body weight and prevent obesity, studies have examined food intake, satiety, body weight, body mass index, and body composition following consumption of different types of oils. Furthermore, dietary fatty acid composition plays an important role in metabolism and gene expression.^76^ Some studies have explored the possible actions of oleic acid-rich oils, such as olive and canola oils, given older data suggesting preferential partitioning of oleic acid for oxidation versus retention in body storage compartments.

A few studies examined the direct actions of canola oil-based diets on human energy metabolism. For example, Maljaars et al.^75^ analyzed the satiating effect of fat and its physiochemical properties in a double-blind, randomized crossover study. In this study, fat emulsions consisting of 6 g of shea, canola oil, or safflower oil were each provided to 15 healthy subjects. After 4 days, the canola and safflower oils significantly increased fullness and reduced hunger compared with the saline control treatment, but no effects on food intake were observed. Gut peptides involved in the regulation of satiety and food intake were also analyzed in this study. Cholecystokinin secretion increased with canola oil feeding compared with the control, but no dietary effect was observed on peptide YY secretion. This study concluded that canola oil can increase cholecystokinin levels, which may, in turn, mediate the satiating effects in ileum, but not in peptide YY. Diaz et al.^77^ assessed the effects of the dietary supplements chromium picolinate and conjugated linoleic acid, with canola oil provided as the placebo, on energy restriction and exercise-induced changes in body weight, body composition, and body mass index in overweight premenopausal women over a period of 12 weeks. Despite the initial hypothesis that women who consumed the dietary supplements would attain lower fat deposition, higher lean mass, and greater positive changes in indexes of metabolism compared to women who consumed a placebo (canola oil), the results of this study supported the assertion that the combination of conjugated linoleic acid failed to influence losses of body weight and fat deposition compared to the placebo canola oil treatment.

Gillingham et al.^78^ tested the effects of a high-oleic canola oil diet on energy expenditure and body composition in hypercholesterolemic subjects. After a 28-day experimental period, no differences were observed in subjects' body composition, resting and postprandial energy expenditure, and substrate oxidation among the groups consuming a high-oleic canola oil or flaxseed-canola blend oil compared with a typical high-fat Western diet. The results of this study suggest that replacement of a typical high-fat Western diet with high-oleic canola oil or flaxseed-canola blend oil does not influence energy expenditure or body composition.

Unfortunately, substantial limitations exist with some studies that have attempted to explore the effects of different dietary fats on energy expenditure and body composition. In most studies, the levels of food and energy consumed by study volunteers were adjusted to avoid weight changes during the feeding period. As such, the risk of bias within studies should be considered because any weight changes during the diet periods cannot explain the efficiency of canola oil on energy metabolism. For instance, Södergren et al.^34^ did not report the adjustment of food intake during the feeding period. This group identified an average of about 1% weight loss and a statistically decreased body mass index after a canola oil-based diet compared to the baseline. However, considering biological variation and flexibility, this kind of weight loss and body mass index reduction may not provide an effective, long-term impact on disease risk.

### Influence of canola oil on insulin sensitivity and glucose tolerance

Evidence has shown that SFA intake is linked with insulin resistance, obesity, and metabolic syndrome.^15^ In a recent review by Gillingham et al.^15^ on the health benefits of MUFAs, it was concluded that MUFAs are capable of favorably modulating insulin sensitivity and glycemic control when substituted for SFAs. Many dietary intervention studies have tested the effects that a substitution of SFAs with MUFAs or PUFAs has on insulin sensitivity.^15^,^79^–^81^ However, many other factors beyond dietary fatty acid composition may influence glucose tolerance and insulin sensitivity. In this arena, the focus has been on the clinical outcomes of canola oil-based diets on insulin sensitivity and glucose tolerance compared to diets higher in SFAs and unsaturated fatty acids.

Uusitupa et al.^35^ reported lower levels of glucose and steeper glucose disappearance rates in participants who consumed a canola oil-based diet compared with a high-SFA diet after an intravenous glucose tolerance test. Södergren et al.^34^ reported that consumption of a canola oil-based diet lowered fasting plasma glucose levels compared with an SFA diet, while fasting insulin levels did not differ between the two diets. Results from Gustafsson et al.^26^ showed that fasting blood glucose levels decreased 6% following a canola oil-based diet compared to baseline values (containing SFA > 15%). No significant differences were found between canola oil and sunflower oil diets. Serum insulin levels were not altered, likely due to large inter-individual variations.^23^ Iggman et al.^38^ conducted a randomized, controlled study in which dairy fat was replaced with canola oil for 3 weeks in hypercholesterolemic participants. No differences were observed between the canola oil- and dairy fat-diet consumption phases, regardless of insulin sensitivity levels and fasting glucose concentrations. However, only the canola oil diet tended (*P* = 0.1) to increase the glucose disappearance rate (33% compared with baseline).^38^ Due to the increased glucose levels often present among subjects with metabolic syndrome,^33^ subjects' conditions in this study from Iggman et al.^38^ may have impacted the regulation of glucose levels throughout the dietary treatment. Compared with partially hydrogenated soybean oil, canola oil showed a lower insulin concentration and lower homeostasis model assessment ratio^41^; however, there was no difference between these two oils with regard to their effects on plasma glucose concentrations, which suggests canola oil may have a potential effect on insulin sensitivity.^41^ In general, canola oil-based diets show positive results in modulating glucose and insulin levels compared with SFA-based diets.

Only a few studies have reported the effects of canola oil-based diets on glucose and insulin levels compared with other vegetable oil-based diets,^41^ mixed oil-based diets,^25^,^82^ or protein-rich diets.^83^ Results failed to show consistency in the effects of canola oil on insulin sensitivity and glucose tolerance. To fill the important gap in knowledge regarding the effects of canola oil on risk factors for type 2 diabetes, researchers continue to explore the effects of diet on glycemic control, fasting blood glucose, and insulin sensitivity in order to promote healthy food choices for the public.

### Canola oil and cancer risk in in vitro and in vivo studies

The association between lipid intake and various cancers has been understudied, particularly in humans. While no human clinical trials specific to canola oil and cancer have been performed to date, in vitro and in vivo animal data exist and are examined here. Jiang et al.^84^ pointed out that omega-3 fatty acids are toxic to tumor cells, but omega-6 fatty acids, such as linolenic acid, enhance carcinogenesis and tumor cell growth. Nevertheless, in a systematic review by Hooper et al.,^85^ no consistent effects of omega-3 fatty acids on the incidence of cancer were identified. Over the past few decades, investigations of the protective effects of dietary fat quality, particularly omega-3 fats, on common cancers, including breast,^86^ colon,^87^ and prostate cancers,^88^ have not been exclusively focused on fish oil.^75^,^89^–^91^ Plant-based oils such as canola oil, which is a good source (11%) of ALA, have also been examined for their potential to modulate cancer cell growth and death. Protective effects of canola oil compared with corn oil against breast and colon cancers have been reported in various cell and animal studies; however, more research is required to provide definitive direction in this area.^76^,^92^–^95^

Certain human studies have investigated the clinical effects of canola oil on carcinogenic substances. Fang et al.^96^ found lower levels of DNA adducts of malonaldehyde in participants consuming a canola oil-based diet compared to subjects consuming a sunflower oil-based diet. This result was not surprising, as sunflower oil would be expected to be high in PUFAs, resulting in enhanced peroxidation of PUFAs compared with the rate of peroxidation in the canola oil group. In a study by Nair et al.,^90^ however, DNA adducts, formed by carcinogenic agents, were not decreased in males but were, on average, 44 times lower in females on a canola oil diet compared to a soybean oil diet.

Wang et al.^97^ designed a human case-control study, which determined that total fat intake was positively associated with breast cancer risk when adjustments were made for age, race/ethnicity, and total energy intake. Also, compared to women cooking only with olive or canola oil, those cooking only with vegetable or corn oil were at 30% increased risk of developing breast cancer. The overarching objective in these studies was to examine the association between dietary fat intake, cooking oil usage, and breast cancer risk in a population-based, multi-ethnic, case-controlled environment. Among the fat components estimated from the food frequency questionnaires, including SFAs and oleic and linoleic acids, oleic acid was more strongly associated with breast cancer than was linoleic acid, whereas SFA intake failed to associate with breast cancer risk. To conclude, studies exploring linkages between canola oil consumption and cancer risk modification remain equivocal.

## Discussion and Future Prospects

**Figure 2:**
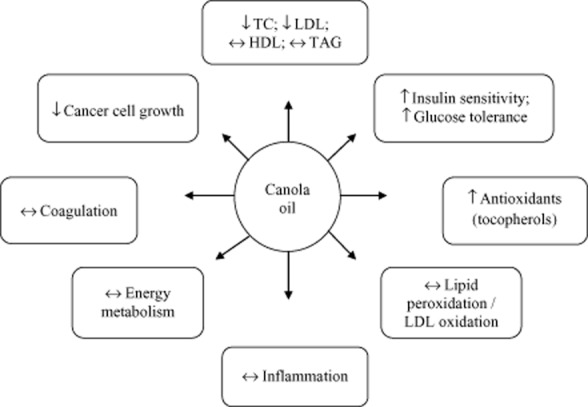
Evidence of the effect of canola oil on health-related risk factors.

Results of this review regarding the biological efficacy of health benefits from canola oil have demonstrated both positive and neutral actions, as illustrated in Figure 2. The ability of canola oil to suppress TC and LDL-C levels compared with SFA or other vegetable oils and the consequent effect on reducing the risk for CHD is well defined, and some of the mechanisms explaining the outcomes of those studies are delineated. In terms of modulating HDL-C and TAG levels, the role of canola oil remains more controversial. High-MUFA oils, such as canola, may offer protection from lipoprotein oxidation. Similarly, data are not unequivocal, but canola oil consumption may be associated with positive immunomodulatory actions compared to the consumption of other oils. In terms of energy metabolism, no clear-cut pattern exists regarding the ability of canola oil to alter energy balance or result in weight reduction; however, some studies suggest that enhancement of fatty acid oxidation may occur for oleic acid compared with other fatty acids. Lastly, provocative recent data suggest that canola oil may have a protective role against certain forms of cancer. More studies are required to better delineate the role of canola oil on health biomarkers.

Despite the primary findings, the present analysis possesses certain limitations. For instance, despite an extensive literature search, the number of valid human interventional studies fulfilling the inclusion criteria remained low. Canola oil was found to be associated with decreased CHD risk biomarkers, which is likely due to its fatty acid composition^13^,^14^,^16^,^24^ and antioxidants.^26^,^73^,^74^,^95^ That said, some evidence exists to suggest that canola oil may exert its biological effects via additive or synergistic actions of its individual components.^98^

The current review sheds light on the benefits of canola oil based on current evidence. Ongoing projects in North America are focusing on molecular mechanisms and cardioprotective effects of canola oil. One multicenter study including four North American universities is currently exploring the cardioprotective effects of canola oil and involves a clinical trial analyzing the influence of canola and flax oils on endothelial function, inflammation, oxidation, body composition, and plasma lipid levels (see http://www.clinicaltrials.gov/ct2/show/NCT01351012?term=canola&rank=4). An additional major objective is to correlate common genetic variants in the fatty acid desaturase 1 and 2 gene cluster with ALA conversion to the longer chain omega-3 fatty acids eicosapentaenoic acid and docosahexaenoic acid. Other research groups are filling important gaps in knowledge regarding the effects of canola oil for managing insulin resistance and improving glycemic control (see http://www.clinicaltrials.gov/ct2/show/NCT01348568?term=canola+oil&rank=3), and the blood vessel function in peripheral arterial disease (see http://www.clinicaltrials.gov/ct2/show/NCT01250275?term=canola+oil&rank=1). Results from these ongoing studies are expected to add to the growing evidence regarding the effects of canola oil on health. Recent findings and recommendations regarding canola oil for human health were also presented at the 10th European Federation for Science and Technology of Lipids Congress meeting.^99^

## Conclusion

After 15 years of continuing research on canola oil since the latest review by Dupont et al.,^2^ evidence shows a number of potential health benefits of canola oil consumption (Figure 2). Canola oil can now be regarded as one of the healthiest edible vegetable oils in terms of its biological functions and its ability to aid in reducing disease-related risk factors and improving health. Current research is expected to provide more complete evidence to support the health-promoting effects of canola oil when consumed at levels consistent with dietary guidelines.
